# Gunshot Wounds of the Abdomen

**Published:** 1874-04

**Authors:** 


*The Nature of Gun-Shot Wounds of the Abdomen, and their Treatment.* By Eugene Peugnet, M. D. New York: William Wood & Co., 1874. Buffalo: H. H. Otis.

This monograph is based upon a review of the case of the late James Fisk, Jr., and is interesting therefore as a medical history of that celebrated case, and the medico-legal questions to which it gave rise.

The author divides his work into five parts, considerering: I. The history of the case of James Fisk, Jr.. II. Description of Shock. III. Penetrating Gunshot Wounds of the Abdomen. IV. The Physiological and Toxicological effects of Morphine. V. The Medical Jurisprudence of the Stokes Case.

Under the first head a concise statement is made of the familiar facts in the case of James Fisk, Jr. Chapter Second gives a description of a shock and the causes which act in its production. The chapter is for the most part, a compilation or quotation from surgical and other writers, and states in a brief manner, what is understood by the term "shock." Under penetrating gunshot wounds of the abdomen, the author gives the statistics of gunshot wounds of the abdomen, and cites a few typical cases of severe penetrating wounds of the abdomen.

The author considers the great danger in these cases to be septicæmia. He considers probing as poor practice, not so much from fear of exciting peritonitis as of renewing the shock, or of displacing the intestines, and thus, if wounded, giving an opportunity for the escape of their contents. He considers the best treatment an expectant rather than an anticipatory one; he therefore considers the administration of opium in continued doses as practiced in the treatment of idiopathic peritonitis as uncalled for, as the danger is more frequently from septicæmia than from peritonitis, and that when peritonitis is present it is self-limited, adhesive and curative.

Chapter Fourth upon the Physiological and Toxical effects of morphine, give a summary of the action of mophia as an opiate, and also its mode of action when administered in over dose. The consideration of the Stokes case closes the book. This portion will be read with interest, as it gives a brief summary of those celebrated trials, and the medical evidence there brought forth. Dr. Peugnet arrives at the following conclusions:

1. The shooting of Fisk was not done in self defence, but with premeditation.

2. The wound in the abdomen was not necessarily fatal, and that the morphine was the immediate cause of death.

We are not prepared to say how far the readers of the work will agree with the author in his conclusions. He seems however, to have carefully drawn his conclusions and stated them upon the evidence presented to his own mind. We have read the work with interest, and should be pleased to see the authors ideas of reform in our way of conducting medico-legal cases, carried out. We notice one or two little peculiarities in the work which we do not understand. If it was written for professional readers, why does the author stop to explain that the ileum is "the small intestine," or that the obdurrator foramen is "an opening between the hip-joint and seat," these may however be explained, by the fact, that the paper was first read before the medico-legal society of New York., a body composed of legal gentlemen as well as of physicians. On the whole the book is well written, and is a valuable acquisition to medical literature.

